# A situational analysis of tobacco control in Ghana: progress, opportunities and challenges

**DOI:** 10.29392/001c.12260

**Published:** 2020-04-03

**Authors:** Arti Singh, Ellis Owusu-Dabo, Noreen Mdege, Ann McNeill, John Britton, Linda Bauld

**Affiliations:** * School of Public Health, KNUST, Kumasi, Ghana; † University of Edinburgh, Edinburgh, UK; ‡ University of York, York, UK; ** King’s College London, London, UK; †† University of Nottingham, Nottingham, UK; ‡‡ University of Edinburgh, Edinburgh, UK

**Keywords:** fctc, mpower, smoking, ghana, tobacco

## Abstract

Tobacco use is the leading cause of preventable deaths in the world, with most of these deaths now occurring in low and middle-income countries (LMICs). Sub-Saharan Africa (SSA) is at an early stage of a tobacco epidemic and is, therefore, particularly vulnerable to rapid growth in tobacco consumption. More than a decade into the implementation of the World Health Organization’s Framework Convention on Tobacco Control (FCTC), State Parties in several countries in SSA, such as Ghana, have yet to fully fulfil their obligations. Despite early ratification of the FCTC in 2004, progress in implementing tobacco control measures in Ghana has been slow and much work remains to be done. The aim of this paper is to critically reflect on tobacco control implementation in Ghana, identify significant research priorities and make recommendations for future action to support tobacco control implementation. We emphasize the need for stronger implementation of the FCTC and its MPOWER policy package, particularly in the area of tobacco taxes, illicit trade and industry interference.

Due to the decline of tobacco use in several high-income countries (HICs), the tobacco industry has sought to expand into low-and middle-income countries (LMICs), particularly in Africa, Asia, and Eastern Europe. ^1^ As a result, it is estimated that if tobacco prevention and control policies are not implemented, smoking prevalence in Africa will increase by over a third by 2030, from 15.8 % in 2010 to 21.9%,^1^ the largest expected regional increase globally. This will result in millions of premature deaths from a range of diseases including non-communicable diseases (NCDs) such as cardiovascular disease, chronic respiratory diseases, cancers and diabetes; and from communicable diseases such as tuberculosis.

The World Health Organization (WHO) has provided guidance for countries to implement and manage tobacco control policies to prevent this epidemic through the Framework Convention on Tobacco Control (FCTC) in 2003. ^2^ The FCTC remains one of the most successfully endorsed treaties with 181 parties (44 in Africa) currently signed up to it, representing 90% of the world’s population. ^3^ Despite this commitment, implementation of effective tobacco control policies and programs in LMICs is lacking, subverted by the tobacco industry who continue to weaken and undermine policy development and execution. ^4^

One of the most effective ways to strengthen tobacco control in LMICs is to address the current evidence gap by creating country-specific and policy-relevant research to aid the development, implementation and evaluation of tobacco control. There are two challenges to achieving this. First, there is the lack of LMIC researchers with appropriate skills, resources and expertise to identify research priorities and conduct the research in tobacco control, particularly given that the health challenges in LMICs have tended to be dominated by communicable diseases. ^3^,^4^ Second, engagement with policymakers and government in LMICs is problematic due to a dearth of researchers in these countries with the necessary substantive and methodological expertise who also have good links with relevant stakeholders. ^4^

To address these challenges the Tobacco Control Capacity Programme (TCCP), a £3.4 million program funded by Research Councils UK as part of the Global Challenges Research Fund (GCRF), is designed to support research institutions in LMICs to develop policy-relevant research in tobacco control with the aim of improving research capacity in LMICs. ^4^ It involves academics from the United Kingdom (UK) who will work alongside research organizations in Bangladesh, Ethiopia, The Gambia, India, South Africa, Pakistan, Uganda, and Ghana. As one of the countries participating in the TCCP, Ghana has an opportunity to develop up to three country-specific research projects with support from researchers in the UK and other LMICs under the themes of tobacco taxation; industry interference and illicit trade. To inform the design of these research projects, the objective of this paper is to critically reflect on tobacco control implementation in Ghana via a situational analysis. First, it will present an overview of the development of tobacco control in Ghana. Next, we consider the extent to which tobacco control policies have been implemented in Ghana. Finally, we identify priorities for future action to support tobacco control implementation in Ghana, which will shape our future research priorities as part of the TCCP program.

## Development of Tobacco Control in Ghana

Ghana has been a world leader in introducing anti-tobacco legislation, having been the first country to prohibit advertising, under a government directive issued as early as 1982. ^5^ Ghana has also been an active member in the development of the WHO FCTC, being one of the first five African countries to become a party to the Convention, and the 39^th^ country to sign the FCTC (on the 20^th^ June 2003), and ratifying the Convention on the 29^th^ November 2004. ^6,7^ Ghana has also performed an active role by chairing committee meetings at the 1^st^ and 2^nd^ Conferences of the Parties, in Geneva (2006) and in Thailand (2007) respectively. It also played a significant role in tobacco control activities and programs worldwide such as the Framework Convention Alliance seminar for non-governmental organisations (NGOs) in the Africa Regional Office (AFRO) region in 2007; the 1^st^ session of the Intergovernmental Negotiating Body on the Protocol on Illicit Trade in Tobacco Products in Geneva in February 2008; and hosting the WHO sponsored Consultation on Regional Capacity Building for Tobacco Control in Africa. ^8,9^

In 2012, the Government of Ghana passed the Public Health Act (851) which includes the Tobacco Control Act that includes measures on smoking in public places; tobacco advertising, promotion, and sponsorship; and tobacco packaging and labelling and others. ^9^ Following the Act in 2012, The Tobacco Control Regulations (L.I. 2247) entered into force on January 4^th^, 2017, which provided 18 months for compliance with public smoking restrictions, among other measures and 18 months for compliance with pictorial health warnings from the date the Food and Drug Authority (FDA) issued the new health warnings electronically. ^10^ A summary of the tobacco control activities in Ghana since the year 2000 is provided in Table 1.

## Implementation of Tobacco Control in Ghana

The WHO introduced the MPOWER measures in 2008 which was set up to expedite the delivery of the WHO Framework Convention on Tobacco Control (FCTC) at a country-wide level (i.e. monitoring tobacco use, protecting people from second-hand smoke, help to quit, warnings about dangers of tobacco, enforce ad bans and raise taxes). These measures are practical, cost-effective ways to scale up country-level implementation of effective interventions to reduce the demand for tobacco that is contained in the WHO FCTC.^11^ Each MPOWER measure corresponds to at least one provision of the WHO Framework Convention on Tobacco Control. In total there are six measures, Ghana’s achievements in advancing each of these measures are now considered.

### (I) Monitoring Tobacco Use

Article 20 of the WHO FCTC calls for countries to conduct surveillance activities to monitor the impact of tobacco use and tobacco prevention programs and policies on their populations. Ghana has no national system for epidemiological surveillance of tobacco consumption and related social, economic and health indicators. ^12^ Ghana has to date conducted four rounds of the Global Youth Tobacco Survey (GYTS); 2000, 2006, 2009 and 2017 and two rounds of the Global School Personnel Survey (GSPS) in 2006 & 2009 and one round of Global Health Professional Students Survey (GHPSS) in 2006. ^8^ The GYTS is a component of the Global Tobacco Surveillance System (GTSS) for systematically monitoring tobacco use (smoking and smokeless) and tracking key tobacco control indicators associated with ages 13-15.^13^ Aside from the GYTS and the GHPSS, tobacco-related data were also included in the Demographic Health Service (DHS) conducted by the Ghana Statistical Services among age groups 15-49 years and till date has been done in 2002/3, 2008 and 2014.^14^

There is scarcity of data on prevalence of tobacco use and the role played by demographic and socioeconomic factors from many countries in the African region including Ghana. Despite the scattered nature of the data, it is evident that the rates of tobacco use are lower in Ghana compared to other countries in the region and globally. Despite this, every year more than 5000 people are killed by tobacco-related disease and more than 5000 children (10-14 years old) and 491000 adults (15+ years old) continue to use tobacco each day in Ghana. ^15^ Although cigarette smoking remains the commonest form of tobacco used, other forms of tobacco are also existent in Ghana; such as pipe smoking (shisha), chewing, sniffing, smokeless tobacco and tawa use among young people.

All studies conducted to date indicate wide gender diversity with male smokers far outnumbering female smokers. ^16–19^ The most recent DHS in 2014 reports the prevalence of cigarette smoking among males to be 4.8% and females 0.1%. ^14,20^ Regional differences in smoking prevalence also exist in Ghana, with several studies demonstrating higher prevalence of tobacco use among those living in remote areas (such as the Northern parts of Ghana); the three regions in the Northern regions have the highest prevalence of tobacco use; 31.2% in Upper East, 22.5% in Northern and 7.9% in the Upper West region.^19,21^ In terms of age groups, 25-34 and 35-59 year-olds have a higher prevalence of cigarette smoking as compared to 15-24 year olds. ^22,23^ Most of the findings of these studies including the DHS national surveys in all the 3 rounds (2002/3, 2008, 2014) are consistent with previous studies in Ghana and elsewhere in Africa where the prevalence of smoking in Ghana is one of the lowest in sub-Sahara Africa.

Among the youth, according to the GYTS (2000 to 2017), cigarette smoking is decreasing; current smokers (smoked cigarettes on one or more days in 30 days preceding the survey) made up 4.2% (2000) but decreased to 2.7% (2006) and increased slightly to 3.6% (2009) and then to 2.8% (2017). ^24^ However, the use of other tobacco products is higher than cigarette use; 14.6% of the sample had used tobacco products other than cigarettes such as chewing tobacco and snuff in 2000, decreased to 10.4% in 2006, then to 10.6% in 2009 and then 6.4% in 2017.^13,24^ Shisha use (water pipe) in particular is indicated to be on the rise and was used among 5.3% of students surveyed in 2017.^13^

### (II) Protect People from Tobacco Use

WHO FCTC Article 8 calls for countries to enact and enforce laws requiring complete smoke-free environments in all indoor public spaces and workplaces. The Tobacco Control Act in Ghana prohibits smoking in “an enclosed or indoor area of a workplace, or any other public place except in a designated area” (partial smoking ban). ^25^ A “workplace and public place” includes “sports stadia and other sports arenas, whether fully enclosed or not.” A public smoking ban was later introduced in 2013, which was then followed in 2016 by the release of the L.I.2247, which included requirements for display of no smoking signs, signs at areas designated for smoking and a right for the owner to prohibit smoking. ^10^ It is recommended that separate and completely enclosed smoking rooms, separately ventilated to the outside, and kept under negative air pressure in relation to the surrounding areas are allowed. However, the difficulty of meeting these requirements set out for such rooms appear to be practically impossible and there is currently no reliable empirical evidence present to ascertain whether they have been constructed. ^26^

Second-hand smoke (SHS) exposure to tobacco smoke is common among young people in Ghana, with 23% and 39% respectively of students reporting exposure to tobacco smoke in the home and in public places. ^13,27^ Although the public smoking ban is fairly comprehensive in its coverage in Ghana, an evaluation of the public smoking ban in hospitality venues pre (2007) and post ban (2015) showed that indoor smoking was observed in 86% and 56% of the facilities respectively with very high SHS exposure as indicated by levels of fine particulate matter 2.5 (PM_2.5_). ^28^ The only intervention shown to completely protect people from the dangers of SHS from tobacco is by establishing environments that are completely smoke-free and do not allow any exceptions (comprehensive smoking ban). ^29^ Therefore the current measures in Ghana to accommodate smoking, such as the presence of designated areas for smoking and ventilation systems (partial smoking ban) do not effectively eliminate second-hand smoke and prevent exposure. ^30,31^

### (III) Offer help to Quit Tobacco Use

Article 14 of the WHO FCTC, which addresses tobacco cessation includes setting up appropriate health care infrastructure, integration of advice into current health-care programs in at-risk populations; availability of free quitlines and mobile cessation services as well as medication and treatment. In 2002, Ghana organized the Quit and Win Cessation competition in collaboration with WHO, Tobacco-free Initiative and the Ghana Health Service. ^32^ The program was aimed at helping adult smokers to quit smoking and to educate non-smokers from initiating smoking. Despite developing a draft manual on cessation in 2007 by the Ghana Health Service, it was only officially published in 2017. ^33^ The Smoking Cessation Clinical Guidelines of Ghana provides information on the various forms of cessation support from brief intervention to the more detailed pharmacological interventions. Nicotine Replacement Therapy (NRT) and smoking cessation services are widely unavailable and also not covered by the National Health Insurance and there are currently no telephone quit lines available for smoking cessation. Nevertheless, some community psychiatric nurses provide limited counseling and cessation services based on substance abuse but not specifically on tobacco dependence treatment. ^34^ With Ghana currently lacking a comprehensive and integrated program on tobacco dependence and cessation and cost of treatment of tobacco dependence and cessation services not covered by the National Health Insurance, it still has a long way to go in addressing the full implementation of FCTC Article 14.

### (IV)) Warnings About the Dangers of Tobacco

**Figure 1:**
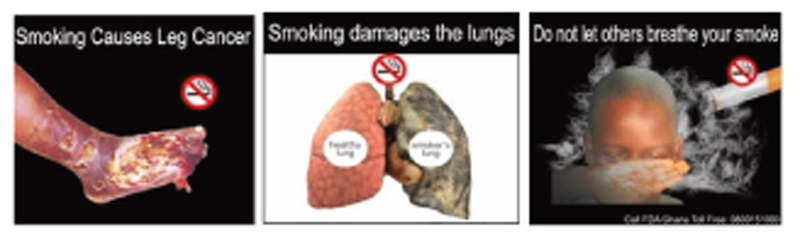
Some examples of pictorial warning labels introduced in Ghana in 2018. (Source: FDA, Ghana).

Article 11 of the WHO FCTC requires Parties to the Convention to implement large, rotating health warnings on all tobacco product packaging and labeling. It is also recommended that Parties should mandate full-colour pictures or pictograms in their packaging and labeling requirements. Ghana’s Public Health Act (857) requires that a primary health warning covers 50% of the front of the pack and a secondary warning cover 50% of the back of the pack. ^7^ In line with completing the requirements of the WHO FCTC Article 11, Ghana, as part of the Legislative instrument (L.I. 2247), introduced pictorial health warnings with its accompanying text covering 50% at the front and 60% at the back, positioned at the lower portion of each of the principal display areas in October 2018 (Figure 1).^10^

### (V)) Enforce Bans on Tobacco Advertising, Promotion and Sponsorship

Article 13 requires Parties to legislate a comprehensive ban on all tobacco advertising, promotion and sponsorship (TAPS), both within the country and that originating from and entering the territory. As early as in 1982, the government in Ghana banned tobacco advertisements on TV, radio and print media. ^35^ This was later followed by the 2012 Public Health Act which also had a section on TAPS. Ghana has since successfully banned tobacco advertisements in all media outlets including all direct tobacco advertising such as television, radio, magazines, newspapers, billboards and the Internet. The National Media Commission’s Guidelines for Broadcasting also stipulates that liquor consumption and smoking should be shown on TV or films only when consistent with plot and character development. There is also a ban on cross-border advertising and there are laws in place for violation of direct advertising bans. ^12^ In 2016 Ghana was recognized as one of the highest achieving countries to enforce bans on tobacco advertising. ^11^ Ghana is also one of the only nine countries in the African Region to have enacted comprehensive anti-TAPS laws. ^8^ In terms of compliance of TAPS bans, Ghana was given 8 points out of 10 for good compliance with the advertising ban on mass media. ^11^ However, evaluation of the impact of the ban and data on violations at point of sales are currently lacking.

### (VI) Raise Taxes on Tobacco Products

Increases in tobacco taxes are widely regarded as a highly effective strategy for reducing tobacco use and its consequences, and the WHO recommends that tobacco cigarette taxes should constitute 75% or more of retail sales price. ^36^ Ghana’s tax administration is unitary and administered by the Ghana Revenue Authority (GRA) through its domestic tax revenue and Customs divisions. ^37^ Over the past 10 years, the tobacco tax structure has changed significantly. Until 2007, Ghana applied a flat ad valorem rate of 140% (of the Cost, Insurance and Freight [CIF]) on all tobacco products. ^8,34,37^ The tax structure was changed to a specific tax regime from ad-valorem for simplicity in administration and enforcement while ensuring a steady stream of revenue and discouraging tax evasion and smuggling in 2007. The tax structure was changed again to impose the flat ad valorem rate of 150% of CIF value in 2010. Although, this seems like a high rate, when converted as a percentage of the retail price, excise tax represents only 22% of the price and the total tax (excise + import duty + VAT + other levies) share is 31% of the retail price, well below WHO’s recommended excise taxes on tobacco products. ^8,37^ Later in 2015, the excise duty rate of tobacco products was further increased from 150 % to 175 % of which excise tax represents about 28% of the price. The excise tax is levied as an ad valorem rate of 175% of the CIF value, but because the CIF value is such a small fraction of the retail price, the excise tax as a percentage of the retail price is well below the average tax burden of middle-income countries and WHO’s recommendation of an excise tax burden of 75% of the retail price. ^38^ The fundamental problem in the tax structure for Ghana is that the base on which the excise tax is levied is small and subject to manipulation by the tobacco industry. ^39^ The FCTC Article 6 recommends that parties implement a simple tobacco tax system by adopting specific mixed excise systems, however, in Ghana, taxes are purely ad valorem thus making it prone to manipulation by the tobacco industry. According to tax simulation modeling studies for LMICs, the tobacco industry has an incentive to raise the net-of-tax price if the excise tax is levied as a specific tax thus enhancing the consumption reducing impact of the tax increase. ^38^ However, if the excise tax is levied as an ad valorem tax (as in the case of Ghana), the industry responds in a way that undermines the increase in the excise tax thereby undermining the public health and fiscal benefits of the tax increase. ^8,37,38^ Therefore, strengthening of tax administration and protecting public health policies from tobacco industry interference (FCTC article 5.3) should be prioritized.

## Future Priorities to Support Tobacco Control Implementation in Ghana

Ghana has made substantial progress in implementing tobacco control measures in line with the FCTC and MPOWER framework but much work still remains to be done in certain areas. It is imperative for LMICs such as Ghana to undertake tobacco control research and identify gaps to provide a sound basis for the development of policies and programs to retrench the devastation brought about by tobacco. Given the present urgency to implement tobacco control programs throughout the developing world, this next section comprises of five policy-oriented actions and research recommendations for tobacco control in Ghana.

## ACTION 1: The Need for Additional Fulfillment of The Fctc Obligations

Aside from the MPOWER indicators discussed above, other areas of significant importance are the FCTC articles 5.3 (industry interference) and 15 (illicit trade) that have not been fully implemented and much work remains in those areas in many LMICs including Ghana.

Ghana like many other countries faces many challenges in implementing Article 5.3. There are currently no clear policies on how to engage with the tobacco industry in protecting public health policy from influence by vested interests and no guidance on the implementation of the guidelines in Article 5.3 in the government. ^8,34^ Despite the absence of tobacco manufacturing in Ghana, tobacco products importers are regularly engaged in lobbying the government to halt increases in tobacco taxation. ^40^ Further, awareness of the Article 5.3 and its guidelines among government officials and key stakeholders is sparse. ^8^ There is also a lack of literature in the AFRO and Ghana specifically to show the areas being influenced by the industry, indicating the need to explore this largely uninvestigated area to make a case for the full implementation of Article 5.3.

Article 15 on illicit trade in tobacco products which requires Parties to adopt and implement measures to ensure “the elimination of all forms of illicit trade in tobacco products, including smuggling, illicit manufacturing and counterfeiting, and the development and implementation of related national law, in addition to sub-regional, regional and global agreements.” ^2^ In Ghana, illicit tobacco products constitute about 20-30% of the market share as estimated by Customs, Excise and Preventive Services (CEPs). ^8^ Likewise, the Euromonitor, whose data is misleading, also estimates illicit trade volumes accounting for almost 24% of total volume sales in 2016 in Ghana. ^41^ This has been linked to the porous borders and high excise duty in comparison to many regional countries, particularly given the tax increases seen recently. ^12^ The estimation of illicit trade in tobacco on the continent of Africa including Ghana is important for three reasons; first, smuggling is substantial and undermines public health efforts to address the upward path of tobacco use on the continent; second, African countries have been found to be particularly susceptible to the loss of customs revenues as a consequence of cigarette smuggling, thereby weakening the already limited capacity of many African governments to achieve broader economic development goals, and lastly, there is recent evidence indicating the involvement of the tobacco industry in illicit trade. ^3,42^

## ACTION 2: The Need for More Local and Comprehensive Data and Research

To fully determine the impact of tobacco use, comprehensive data on the prevalence of tobacco use and its determinants among the different age categories are required at the country and regional level in Ghana. The lack of locally relevant evidence and data gathering/surveillance and infrastructure as well as inadequate expertise in relation to some areas of research, health systems development, and policy formation and implementation is a critical situation in Ghana. Moreover, as the use of other tobacco products such as shisha increases among the young population, data on the determinants of its use are urgently required to contribute to a holistic tobacco control argument.

## ACTION 3: Build Capacity for A National Action Plan

Although there is a national coordinating mechanism and tobacco control policies, there is a need to mobilize resources (financial and human) for national tobacco control programs with the participation of nongovernmental organizations and the private sector. In view of the multisector character of the Convention, there is a need to promote the role and full engagement of different branches of government in supporting its implementation. This could take the form of constant stakeholder engagement to educate policymakers on the current and future challenges of tobacco control in an effort to address the gap in research and implementation. There should also be designated persons for establishing a system for surveillance, particularly monitoring and evaluation of tobacco control policy interventions as well as tobacco industry mapping and monitoring including developing the administrative capacity of tax and customs departments to counter the illicit trade of tobacco in Ghana.

## ACTION 4: Enforcement of Policies

The emerging battlefronts in smoke-free policies are in the areas related to their implementation and enforcement possibly due to the added distinct challenges of policy fatigue and additional resource constraints. Dedicating funds for enforcement, regular monitoring of public places and designing a system that requires an investigation after complaints may provide some solution to the problem of lack of enforcement and compliance with these policies. Another area that requires the enforcement of policies is the illicit tobacco trade; customs officials need to adopt sophisticated methods to intercept consignments of smuggled tobacco. Additional efforts to curb illicit tobacco trade in Ghana is by increasing international technical support and co-operation and exchange of expertise in Ghana.

## ACTION 5: Behavior Change and Public Awareness

Behavior change communication including public awareness campaigns is essential to control measures to begin the process of changing both attitudes and behavior about smoking and tobacco use, particularly among young people. Health promotion methods such as campaigns can be organized in public spaces such as schools as well as on the media such as radio and television to promote a beneficial healthy and attractive non-smoking image.

## Discussion

Despite having a low prevalence of tobacco use in Ghana, this situational analysis demonstrates reasonable progress in establishing a regulatory framework for tobacco control. However, areas including assessment of the uptake of all forms of tobacco smoking (such as shisha use among young people) and the development of gender-specific and age-specific interventions to reduce current levels and minimize uptake in the future need to be prioritized. Furthermore, assessing the effectiveness of current smoke-free policies also need to be considered high on the tobacco research agenda for Ghana. Other areas such as tobacco taxation, illicit trade, and industry interference also require urgent attention. In this respect, as well as aiding the development of regional networks and policy-relevant research in LMICs, the research and capacity building activities within the Tobacco Control Capacity Programme (TCCP) are organized around addressing these research gaps and have the potential to lead to more rapid generation of subsequent research, informed by inputs from stakeholders. Country-specific areas under the TCCP (Ghana) aim to review the illicit tobacco trade in Ghana, evaluate the current tobacco tax structure and apply tax simulation modeling to predict the optimal taxation system for Ghana and lastly assess smoke-free policies in Ghana. Addressing these priority areas in Ghana is a step towards protecting its population from the destructive health, social, environmental and economic ramifications of tobacco consumption and exposure to tobacco smoke and averting a tobacco-related epidemic in Ghana.

## Figures and Tables

**Table 1:** Roadmap of tobacco control efforts in Ghana (2000-2018)

**Year**	**Tobacco control efforts in Ghana**
1982	Ban on tobacco advertising
2000	Ghana joined the Global Tobacco Surveillance System (GYTSS). 1st Global Tobacco Youth Survey was conducted.
2002	Ghana became a member of the Quit and Win International Smoking Cessation Program and the Ghana National Tobacco Control Steering Committee was established.
2003	1. The National Steering Committee drafted the first National Tobacco Control Bill for approval.
	2. A Demographic and Health Survey for assessing tobacco use among adults.
2004	Ghana signed and ratified the FCTC
2005	1. 2nd National Global Youth Tobacco Survey conducted.
	2. Tobacco Bill re-drafted to reflect FCTC provisions for approval.
2006	1. The Ministry of Health issues a directive to ban smoking in all Ministry of Health facilities.
	2. The Ministry of Transportation also issued a directive to ban smoking in public and private commercial transport.
	3. British American Tobacco closed down its manufacturing company and relocates to Nigeria.
2007	1. The Ministry of Health issued another directive to compel all importers of tobacco products to register their products and comply with the FDA regulatory requirements.
	2. The Ministry, the Ghana National Tobacco Control Steering Committee (GNTCSC) and the Ghana Tourist Board reached voluntary agreements with owners of hospitality areas to create smoke-free areas for non-smokers.
2008	1. Ghana Demographic Health Surveys (GDHS) conducted another survey to determine the national tobacco prevalence among adults.
	2. The GNTCSC reached an agreement with importers to disclose the content of tobacco products to be imported in Ghana.
2009	1. GYTS conducted another survey to determine tobacco use among the youth.
	2. The Ministry of Education developed a School Health Education Policy to promote a healthy lifestyle and prohibit tobacco use by children in educational settings.
	3. FDA directed all tobacco products imported into Ghana to have approved health warnings and cover the required sizes.
2010	Ghana hosted the 2^nd^ meeting of the Working Group of the FCTC Article 17 and 18
2011	The Ministry of Health issued an additional directive to mandate the posting of no-smoking signs on the premises of all health facilities.
2012	In October 2012, Ghana adopted a Public Health Act (Act 851 2012) whose provisions in Part 6 are on Tobacco Control Measures.
2014	GDHS conducted in Ghana
2015	Gha The parliament of Ghana approved an increase in tobacco taxes from 150% of the ex-factory to price to 175% passage of the Excise Duty (Amendment) Bill.
2016	Passage of the Tobacco Control Regulations 2016, (L.I 2247) on compliance with pictorial warnings and smoke-free laws in Ghana
2018	Introduction of Pictorial Health Warning and emission statements on tobacco product packages by the FDA, Source document given to tobacco industries for enrollment in 2018.

FDA - Food and Drugs Authority, FCTC - Framework Convention for Tobacco Control, GDHS - Ghana Demographic Health Survey, GNTCSC - Ghana National Tobacco Control Steering Committee, GYTS -Global Youth Tobacco Survey, GYTSS - Global Tobacco Surveillance System, L.I. 2247 - Legislative Instrument 2247
